# The Roles of Electronic Health Records for Clinical Trials in Low- and Middle-Income Countries: Scoping Review

**DOI:** 10.2196/47052

**Published:** 2023-11-22

**Authors:** Jiancheng Ye, Shangzhi Xiong, Tengyi Wang, Jingyi Li, Nan Cheng, Maoyi Tian, Yang Yang

**Affiliations:** 1 Weill Cornell Medicine New York, NY United States; 2 Northwestern University Feinberg School of Medicine Chicago, IL United States; 3 The George Institute for Global Health, Faulty of Medicine and Health University of New South Wales Sydney Australia; 4 Global Health Research Centre Duke Kunshan University Kunshan China; 5 School of Public Health Harbin Medical University Harbin China; 6 School of Basic Medicine Harbin Medical University Harbin China; 7 The First Affiliated Hospital of Harbin Medical University Harbin China; 8 School of Public Health Shanghai Jiao Tong University School of Medicine Shanghai China

**Keywords:** electronic health records, clinical trials, low- and middle-income countries

## Abstract

**Background:**

Clinical trials are a crucial element in advancing medical knowledge and developing new treatments by establishing the evidence base for safety and therapeutic efficacy. However, the success of these trials depends on various factors, including trial design, project planning, research staff training, and adequate sample size. It is also crucial to recruit participants efficiently and retain them throughout the trial to ensure timely completion.

**Objective:**

There is an increasing interest in using electronic health records (EHRs)—a widely adopted tool in clinical practice—for clinical trials. This scoping review aims to understand the use of EHR in supporting the conduct of clinical trials in low- and middle-income countries (LMICs) and to identify its strengths and limitations.

**Methods:**

A comprehensive search was performed using 5 databases: MEDLINE, Embase, Scopus, Cochrane Library, and the Cumulative Index to Nursing and Allied Health Literature. We followed the latest version of the PRISMA-ScR (Preferred Reporting Items for Systematic Reviews and Meta-Analyses extension for Scoping Reviews) guideline to conduct this review. We included clinical trials that used EHR at any step, conducted a narrative synthesis of the included studies, and mapped the roles of EHRs into the life cycle of a clinical trial.

**Results:**

A total of 30 studies met the inclusion criteria: 13 were randomized controlled trials, 3 were cluster randomized controlled trials, 12 were quasi-experimental studies, and 2 were feasibility pilot studies. Most of the studies addressed infectious diseases (15/30, 50%), with 80% (12/15) of them about HIV or AIDS and another 40% (12/30) focused on noncommunicable diseases. Our synthesis divided the roles of EHRs into 7 major categories: participant identification and recruitment (12/30, 40%), baseline information collection (6/30, 20%), intervention (8/30, 27%), fidelity assessment (2/30, 7%), primary outcome assessment (24/30, 80%), nonprimary outcome assessment (13/30, 43%), and extended follow-up (2/30, 7%). None of the studies used EHR for participant consent and randomization.

**Conclusions:**

Despite the enormous potential of EHRs to increase the effectiveness and efficiency of conducting clinical trials in LMICs, challenges remain. Continued exploration of the appropriate uses of EHRs by navigating their strengths and limitations to ensure fitness for use is necessary to better understand the most optimal uses of EHRs for conducting clinical trials in LMICs.

## Introduction

Clinical trials are a crucial element in advancing medical knowledge and developing new treatments by establishing the evidence base for safety and therapeutic efficacy [1]. However, the success of these trials depends on various factors, including trial design, project planning, research staff training, and adequate sample size [2]. It is also crucial to recruit participants efficiently and retain them throughout the trial to ensure timely completion [3].

Randomized controlled trials (RCTs) are considered the gold standard for evaluating the benefits and risks of health care treatments. Despite their high level of evidence, RCTs are often time consuming and expensive and may be limited by strictly standardized research settings that can hinder the generalizability of their results [4]. One promising solution to this challenge is the use of electronic health records (EHRs) to conduct large and pragmatic trials [5]. However, the gap in health care resources between high-income countries (HICs) and low- and middle-income countries (LMICs) varies greatly [6,7]. Although HICs have made significant progress in using EHR for clinical trials [8-10], little is known about the effectiveness of similar applications in LMICs [11,12]. Understanding the progress made in LMICs and how EHR has been applied to clinical trials can provide valuable insights for promoting and improving population health. Conducting clinical trials in LMICs can also provide a comprehensive evaluation of interventions in different settings beyond HICs.

This scoping review aims to comprehensively understand the roles of EHRs in the life cycle of clinical trials, determine how EHRs were implemented in clinical research settings, and further describe specifically how this technology should be used to support different types of clinical trials in an LMIC context.

## Methods

This scoping review followed the latest version of the PRISMA-ScR (Preferred Reporting Items for Systematic Reviews and Meta-Analyses extension for Scoping Reviews) guideline for the entire review process [13].

### Data Sources and Search Strategy

A comprehensive search was performed using 5 databases for articles published until the end of 2021: PubMed or MEDLINE, Embase, Scopus, Cochrane Library, and the Cumulative Index to Nursing and Allied Health Literature. We prepared the search terms using the patients, problem, or population; issue of interest or intervention; comparison, control, or comparator; outcome; and study type (PICOS) approach. As the search aimed to be as comprehensive as possible and correspond to the research questions, 3 domains including EHRs, clinical trials, and LMICs (based on the list on the World Bank definition) were used to develop the search strategy [14]. A combination of keywords and controlled vocabulary terms related to the target concepts was used. The search strategy was designed and developed by 2 authors (JY and SX) independently and confirmed with an experienced librarian. Multimedia Appendix 1 presents the search strategy.

### Study Selection

Studies were included in this review if they met the following criteria: (1) clear indication of clinical trials; (2) EHR was involved in the trial conduct, including identification, recruitment, informed consent collection, implementation, outcome adjudication, and outcome verification; (3) the study was conducted in an LMIC; (4) the study was published until December 31, 2021; and (5) no language restrictions. The exclusion criteria were as follows: (1) absence of a clinical trial focus; (2) the primary research question was nonclinical (ie, cost analysis study); (3) not available in full text; (4) conference abstracts or posters; (5) nonresearch articles (ie, perspectives, commentaries, letters, and reviews); and (6) retrospective secondary data analysis in a clinical trial, for example, studies that used retrospective data for 2 groups of patients who received different treatments and compared their outcomes.

First, duplicate articles were eliminated from the retrieved articles. Then, 4 reviewers (JY, SX, TW, and YY) independently screened articles based on titles and abstracts to identify the studies that could potentially fit the research question and meet the eligibility criteria. Records were excluded if they were marked as irrelevant by 2 reviewers. For records that were kept or were difficult to decide based on the title or abstract, the full text was scrutinized. When disagreements regarding study inclusion occurred between the 2 reviewers, a third or fourth reviewer was involved in the discussion until consensus was reached.

### Data Extraction

A data extraction form was developed for data extraction. For each included study, we first extracted the studies’ basic information, including the first author’s name, publication year, country, trial setting, trial design, target population, intervention, and outcome. Of note, for trial designs, we considered individual RCT, cluster RCT, quasi-experimental studies, and feasibility pilot studies. To determine how the studies used EHR in conducting the trials, we extracted information on the roles that EHR played at any step in each of the included studies.

### Data Synthesis and Analysis

We conducted a descriptive analysis on the basic information of each included paper and conducted qualitative synthesis to analyze the roles that EHR played in conducting the trials and to identify their associated implications. In the qualitative synthesis process, we referred to an established framework from a publication in 2019 [11]. The study reviewed the current and prospective uses of EHR in clinical trials worldwide and outlined five steps in which EHR could be used: (1) patient identification and recruitment, (2) participant consent and randomization, (3) intervention, (4) outcome assessment, and (5) extended follow-up [11]. On the basis of this framework, we first attempted to map our identified roles of EHR in clinical trials into these 5 steps, and then, we performed modifications by adding our identified new roles of EHR from the included studies. The identifications and articulations of new roles were based on research team discussions until consensus was reached (JY, SX, and YY). In addition, when available, we further synthesized text information about implications of using EHR in conducting clinical trials, by summarizing them as “strengths” and “limitations” under each role of EHR.

### Quality Assessment

We followed the National Heart, Lung, and Blood Institute’s Study Quality Assessment Tools for the quality assessment of the included studies [15]. For studies with control groups, a total of 14 questions were considered, including the adequacy of randomization, blinding of treatment assignment and outcome assessment, use of intention-to-treat analysis, and sufficiency of the sample size. For the quasi-experimental studies without independent control groups (eg, pre-post studies), a total of 12 questions were considered, including clarity in study objectives, participant eligibility criteria, prespecification of outcomes and subgroups, and sample size sufficiency. We marked studies that met ≥80% of applicable criteria as “good quality,” 60% to 80% as “fair quality,” and <60% as “poor quality.”

## Results

### Selected Characteristics of the Included Studies

**Figure 1:**
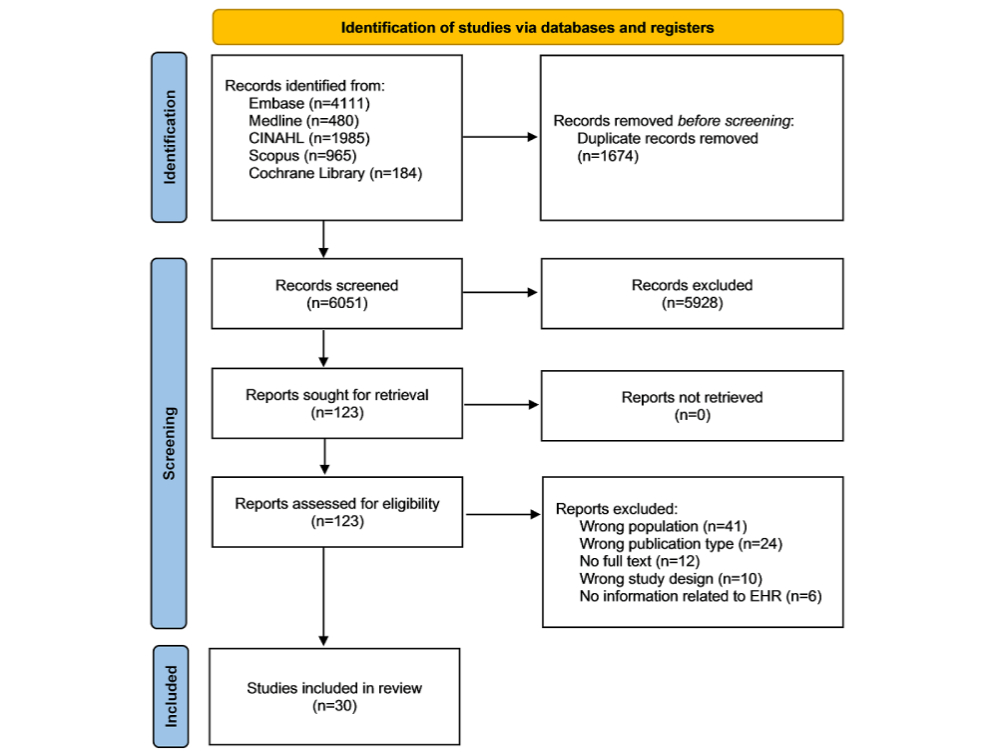
The PRISMA (Preferred Reporting Items for Systematic Reviews and Meta-Analyses) flow diagram of the included studies in the review. EHR: electronic health record.

Figure 1 displays the PRISMA (Preferred Reporting Items for Systematic Reviews and Meta-Analyses) flow diagram for article selection. The initial search from the 5 databases yielded a total of 7725 references. After removal of duplicates, the first round of screening excluded 6051 references for ineligibility, leaving 123 references for full-text screening. A total of 93 references were then further excluded, primarily for wrong populations (ie, studies conducted exclusively in HICs) and wrong study types (ie, study types other than clinical trials). Finally, 30 studies were included in the data charting and analysis.

As shown in Table 1, the 30 studies were conducted in 15 LMICs, with China being the most represented (n=7, 23%), followed by Kenya (n=5, 17%). Zambia, South Africa, and Malaysia were all included in an equal number of studies, with each country being part of 3 (10%; 3 for each) studies. Of note, 27 (90%) of the 30 studies were conducted in a single LMIC, and 3 (10%) were conducted in multiple LMICs [16-18]. Table 2 presents the summaries of the characteristics of included studies. Most of the reported trials were conducted after 2010 (29/30, 97%), with the majority published in 2018 (5/30, 17%), 2019 (5/30, 17%), and 2020 (7/30, 23%). The oldest study was conducted in 2004 (1/30, 3%), whereas the most recent studies were conducted in 2021 (3/30, 10%). For study types, most included studies were RCTs (13/30, 43%), followed by quasi-experimental studies (12/30, 40%), cluster RCTs (3/30, 10%), and feasibility pilot studies (2/30, 7%). Of note, 9 quasi-experimental studies adopted a retrospective design using past data from the EHR systems. Regarding disease types, most studies addressed infectious diseases (15/30, 50%), with 40% (12/30) of them addressing HIV or AIDS. Another 12 studies focused on noncommunicable diseases, such as hypertension, diabetes, cancer, and mental illness. Three studies focused on injuries, such as hip fracture and brain injury.

**Table 1:** Characteristics of the included studies.

Study, year	Country	Trial design	Disease areas	Summary of interventions	Outcomes
Figar et al [19], 2004	Argentina	Quasi-experimental study	Hypertension	A complex antihypertensive intervention program for physicians of the older adults	Systolic and diastolic blood pressure and the proportion of patients who were well controlled
Lakkis et al [20], 2011	Lebanon	Randomized controlled trial	Cancer	An informative SMS text remainder about mammogram tests	Performance of (whether or not performed) the mammogram test
Were et al [21], 2013	Kenya	Randomized controlled trial	AIDS	A CDSS^a^ with clinician-targeted computer-generated reminders	The number of clinical visits before the completion of overdue tasks, including HIV testing, laboratory monitoring, initiating antiretroviral therapy, and making referrals
Li et al [22], 2014	China	Quasi-experimental study, using retrospective data	Cardiovascular disease	Salvianolate injection treatment over 14 days	Indexes of liver and kidney function, including alanine aminotransferase, aspartate aminotransferase, creatinine, and blood urea nitrogen
Zhang et al [23], 2014	China	Quasi-experimental study, using retrospective data	Urologic diseases	Pharmacist interventions that included real-time monitoring of medical records and controlling the prescription of prophylactic antibiotics	Rational use of antibiotic prophylaxis, including frequency of prophylactic antibiotic use, indications for and rate of prophylactic antibiotic use, medical cost/patient, inappropriate prophylactic antibiotic use, rate of correct antibiotic administration, and cost-benefit
Ghadieh et al [24], 2015	Lebanon	Randomized controlled trial	Bacterial infection	A set of reminders to invite participants to get the PPSV23^b^ vaccine	The vaccine administration rate in the clinics
Ali et al [16], 2016	India and Pakistan	Randomized controlled trial	Diabetes	A multicomponent quality improvement strategy	Primary: the proportion that achieves multiple care targets Secondary: achieving individual risk factor targets, mean risk factor changes, and patient reported outcomes (eg, health-related quality of life and treatment satisfaction scores)
Oluoch et al [25], 2015	Kenya	Cluster randomized controlled trial	AIDS	A CDSS with pop-up information and reminder whenever action is needed for an individual patient, and an alert when a patient had immunological treatment failure	Primary: the difference between groups in the proportion of patients who experienced immunological treatment failure Secondary: the effect of CDSS on time from detection of immunological treatment failure to clinical action and time from antiretroviral treatment initiation to first CD4^c^ cell measurement
Wang et al [26], 2016	China	Quasi-experimental study, using retrospective data	Hypertension	A guideline for hypertension management	The incidence of stroke
Al-Hashar et al [27], 2018	Oman	Randomized controlled trial	Chronic disease	Medication reconciliation on admission and discharge, medication review, bedside medication counseling, and take-home medication list	Primary: percentage of preventable adverse drug events Secondary: rates of readmission, rates of emergency department visits, rates of unplanned visits to hospitals or health centers, and the 3 combined
George et al [28], 2018	Kenya	Randomized controlled trial	AIDS	A SMS intervention promoting the availability of oral self-administered HIV self-testing kits	HIV testing rates
Mody et al [29], 2018	Zambia	Quasi-experimental study, using retrospective data	AIDS	A new HIV treatment guideline	Timely antivirus treatment initiation, retention in care at 6 mo, and being retained and on antiretroviral treatment at 6 mo
Bachmann et al [30], 2019	Brazil	Cluster randomized controlled trial	Asthma or chronic obstructive pulmonary disease	A guide and training program for doctors and nurses	Primary: composite scores of treatment changes, spirometry, and new asthma and COPD^d^ diagnosis rates Secondary: the disaggregated treatment and spirometry components of asthma and COPD scores, prescriptions (eg, support tobacco cessation, depression), diseases diagnosed for the first time (eg, cardiovascular disease and diabetes mellitus), and cardiovascular risk assessed
Engelbrecht et al [31], 2018	South Africa	Quasi-experimental study, using retrospective data	Mental illness	A mental health therapy in an occupational therapy-led day treatment center	Hospitalization days, frequency of attendance and admissions to hospital, frequency of attendance and number of days spent in hospital, and attendance rate at day treatment center
Ismail et al [32], 2019	Saudi Arabia	Quasi-experimental study	Renal disease	A patient-centered pharmacist care in the hemodialysis unit, using comprehensive medication review through medication therapy management and motivational interviewing	Primary: changes in serum phosphate levels and differences in number of medications Secondary: systolic blood pressure, serum low-density lipoprotein levels, glycosylated hemoglobin levels, the prevalence and types of medication-related problems, and the rates of therapeutic interventions acceptances or rejections
Kelvin et al [33], 2018	Kenya	Randomized controlled trial	AIDS	An intervention consisting of sending a text message and offering a brief demonstration of the self-testing kit on the site	HIV testing rates
Lima et al [34], 2018	Brazil	Quasi-experimental study, using retrospective data	Cancer	A guideline for hemodynamic and depth of anesthesia monitoring	Postoperative outcomes including the use of cardiac output, central venous oxygen saturation, depth of anesthesia monitoring, intraoperative total fluid volume and colloid volumes, number of patients receiving colloids and received inotropes, rates of postoperative delirium and urinary tract infection, postoperative morbidity, and length of hospital stay
Phillips et al [35], 2020	South Africa	Randomized controlled trial	AIDS	A maternal and child health service in the antenatal clinic through cessation of breastfeeding	Primary: a composite of female participants’ retention in HIV care and viral suppression preceding the long-term adherence and care engagement study visit Secondary: current use of family planning, pregnancies since the trial, maternal hospitalizations and tuberculosis diagnoses in the past year
Tay et al [36], 2019	Malaysia	Quasi-experimental study	Bacterial infection	An education program for both physicians and patients on the rational use of antibiotics for upper respiratory infection and acute diarrhea	Antibiotic prescription rate and rates of reattendance or hospital admission
Wu et al [37], 2019	China	Quasi-experimental study, using retrospective data	Hip fracture	A comanagement program involving both orthopedic surgeons and geriatricians embedding in a pathway of care spanning emergency department presentation to discharge from hospital	Primary: the proportion of patients who received surgery within 48 h of admission to a ward Secondary: the proportion of patients who were admitted to a ward within 4 h of presentation to emergency department, who developed a pressure ulcer, who received geriatrician care, and who received osteoporosis and falls prevention assessment
Ali et al [38], 2020	India	Randomized controlled trial	Depression and diabetes	A multicomponent quality improvement strategy with nonphysician care coordinators and decision support EHRs^e^	Primary: between-group difference in Symptom Checklist Depression Scale scores and a reduction in glycosylated hemoglobin, systolic blood pressure, or low-density lipoprotein Secondary: percentage of patients who met treatment targets or had improvements in individual outcomes; percentage of patients who met all glycosylated hemoglobin, systolic blood pressure, or low-density lipoprotein targets; and mean reductions in Symptom Checklist Depression Scale scores and Patient Health Questionnaire-9 scores
Yang et al [39], 2020	China	Randomized controlled trial	Orthognathic disease	A hydroactive dressing on the nasal ala of patients undergoing orthognathic surgery	The incidence of nasal ala pressure injury associated with nasotracheal intubation
Puttkammer et al [40], 2020	Haiti	Feasibility pilot study	AIDS	An EHR-based alert for adherence intervention	HIV viral load status, antiretroviral treatment adherence, and proportion of patients who were never >7 d late for an antiretroviral treatment refill pickup
Roy et al [41], 2020	Zambia	Cluster randomized controlled trial	AIDS	Adherence club group intervention to improve on-time drug pickup and retention in HIV care through off-hours facility access and pharmacist-led group drug distribution	Primary: time to first late drug pickup Secondary: medication possession ratio, implementation outcomes (adoption, acceptability, appropriateness, feasibility, and fidelity), and viral load suppression at 12 mo
Seth Kalichman et al [42], 2020	South Africa	Randomized controlled trial	Sexually transmitted infections	Two counseling sessions for brief risk reduction sexual behavior change and brief enhanced partner notification	Primary: return sexually transmitted infection visits after counseling and rate of scale representing the percentage of times condoms were used Secondary: sexually transmitted infection risk and prevention-related knowledge assessed by 4 heterogeneous items, HIV stigma assessed by the HIV Stigma Scale, and prevention skills self-efficacy
Mahmood et al [17], 2020	United Kingdom and Malaysia	Randomized controlled trial	Traumatic brain injury	A tranexamic acid treatment	Primary: the volume of intraparenchymal hemorrhage after randomization Secondary: presence of progressive intracranial hemorrhage, new intracranial hemorrhage, presence of cerebral infarction, composite poor outcome, and intracranial hemorrhage volume
Mody et al [43], 2021	Zambia	Quasi-experimental study, using retrospective data	AIDS	A new national HIV treatment	Primary: rates of both antiretroviral treatment initiation and retention on antiretroviral treatment across subgroups in 3 timestamps, antiretroviral treatment initiation within 1 mo of enrollment, and retention in care on antiretroviral treatment at 12 mo
Semeere et al [18], 2021	Uganda and Kenya	Feasibility pilot study	AIDS	A rapid case ascertainment measurement shortly after diagnosis	Performance of (whether or not performed) rapid case ascertainment
Xu et al [44], 2021	China	Quasi-experimental study, using retrospective data	Bacterial infection	A pharmacist-led intravenous to oral antibiotic conversion practice with computerized reminders	Primary: the proportion of patients who converted to oral therapy on the day patients were eligible for the conversion Secondary: length of IV^f^ antibiotic therapy days, total length of antibiotic therapy days, and length of hospital stays
Abdulrahman et al [45], 2017	Malaysia	Randomized controlled trial	AIDS	Mobile phone reminders based on SMS, telephone call, and peer counseling	Primary: improved scheduled clinic attendance and medication adherence self-report Secondary: immunological, virological, and clinical measurements

^a^CDSS: clinical decision support system.

^b^PPSV23: Pneumococcal Polysaccharide Vaccine.

^c^CD4: cluster of differentiation 4.

^d^COPD: chronic obstructive pulmonary disease.

^e^EHR: electronic health record.

^f^IV: intravenous therapy.

**Table 2:** Summaries of the characteristics of the included studies (N=30).

Characteristics		Studies, n (%)
**Year of publication**		
	2017 or before	10 (33)
	2018-2019	10 (33)
	2020-2021	10 (33)
**Trial design**		
	Randomized controlled trials	13 (43)
	Cluster randomized controlled trials	3 (10)
	Quasi-experimental studies	12 (40)
	Feasibility pilot study	2 (7)
**Trial settings**		
	Primary health care	8 (27)
	Secondary health care	3 (10)
	Tertiary health care	8 (27)
**Roles of EHR^a^**		
	Identification and recruitment	12 (40)
	Participant consent and randomization	0 (0)
	Baseline information collection	6 (20)
	Intervention	8 (27)
	Fidelity assessment	2 (7)
	Primary outcome assessment	24 (80)
	Secondary outcome assessment	13 (43)
**Disease areas**		
	Infectious (AIDS) and maternal and perinatal conditions	15 (50)
	Noncommunicable diseases (eg, diabetes, hypertension, cancer, and mental illness)	12 (40)
	Injury	3 (10)

^a^EHR: electronic health record.

### Interventions and Outcomes in Included Trials

The included clinical trials covered various types of interventions, ranging from single behavioral interventions such as informative SMSs for clinical appointments or vaccinations and clinical therapies such as tranexamic acid treatment or Salvianolate injection to complex multifaceted intervention packages for service quality improvements or education programs [26,29,34,43]. Corresponding to the diversity of interventions, the included studies also focused on a wide range of outcomes, including individual clinical outcomes such as blood pressure and incidence of stroke, individual behavioral outcomes such as medication use and retention in antiretroviral care, and facility-level administration data such as the number of hospital visits and vaccine administration rates.

**Figure 2:**
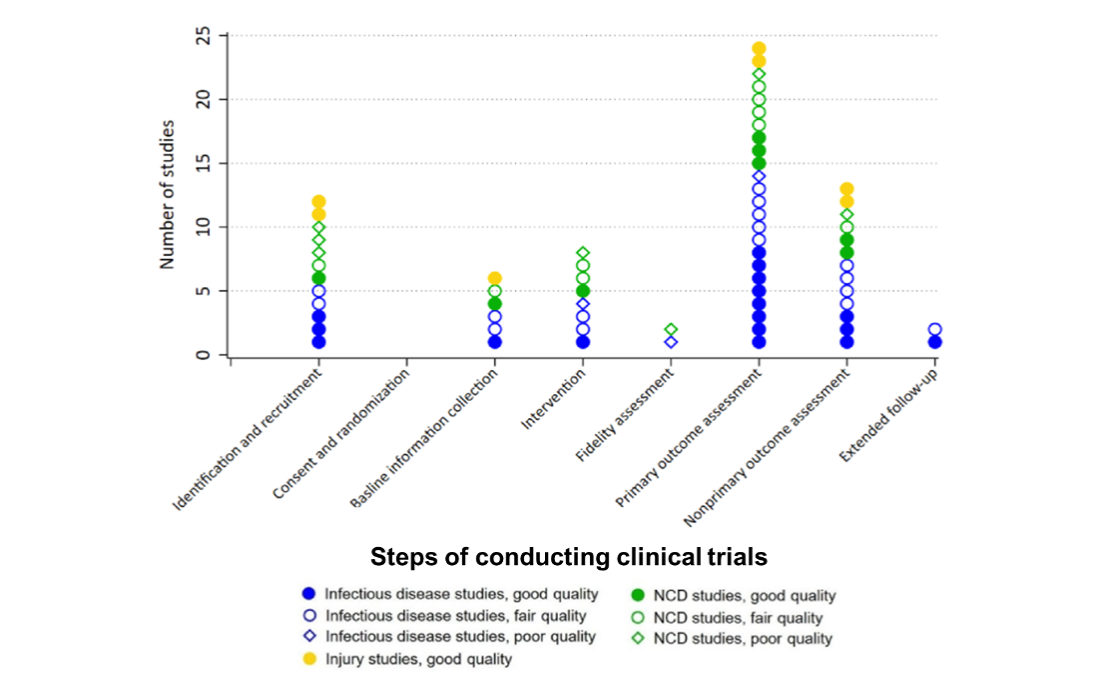
The roles of electronic medical records in the different steps of conducting clinical trials among the included studies. NCD: noncommunicable disease.

Of the 8 steps of conducting clinical trials (Figure 2), we identified 7 in which EHR was used in the 30 included studies, with the only exception for “participant consent and randomizations.” The role of EHR in primary outcome assessment (24/30, 80%) was the most commonly documented, followed by the use of EHR to assess nonprimary outcomes (13/30, 43%). For example, 1 study focused on the effects of adherence club groups on the on-time antiretroviral drug pickup among people with HIV or AIDS, whose primary outcome was “time to first late drug pickup,” and secondary outcome was “the proportion of time that a patient has antiretroviral drug in their possession over 12 months,” and both of them were ascertained using EHR data [41]. One study used EHR to obtain safety outcomes as a nonprimary outcome, which was the adverse change in participants’ bioindicators after the intervention [22]. We summarized that there were 3 main types of information collected from the EHR: people’s clinical information (eg, systolic and diastolic blood pressure), behavioral information (eg, medication use), and health facility administration data (eg, frequency of hospital visits).

### Roles of EHRs in Trial Steps

Table 3 demonstrates the roles of electronic medical records in the different steps of conducting clinical trials among included studies. A total of 12 trials used EHR to identify and recruit study participants based on automatically or manually extracted data. These studies queried a series of data entries of clinical information from the databases to identify their target populations and retrieved the contact information to approach the potential patient participants. For example, Bachmann et al [30] identified eligible participants with asthma or chronic obstructive pulmonary disease using the International Classification of Disease diagnostic codes in a consolidated municipal EHR database in Brazil. Lakkis et al [20] used EHR to extract cell phone numbers of female participants who were recommended to undergo a screening mammogram for breast cancer.

**Table 3:** The roles of electronic medical records in the different steps of conducting clinical trials among included studies.

Roles of EHR^a^ (number of studies)	Descriptions	Implications	References
Participant identification and Recruitment (n=12)	People’s demographic, clinical, and contact information from the EHR systems was either automatically or manually extracted to help researchers identify and then recruit trial participants.	Strength: EHR enabled researchers to access participants that were otherwise unfeasible to access. Strength: EHR enabled researchers to identify and include all eligible patients whose interaction with health systems were electronically recorded, which increased generalizability and sample size at minimal cost. Limitation: some eligible participants may not be documented in the EHR systems. Limitation: artifacts, errors, and misclassifications in EHR may cause inclusion of ineligible participants or exclusion of eligible ones.	[17,19,20,22-26,28,30,33,37]
Baseline information collection (n=6)	For people who were enrolled, many trials used EHR systems to collect their baseline basic characteristics, including demographical and socioeconomical information, which were used as covariates in later analyses.	Not mentioned.	[29,30,32,39,41,43]
Intervention (n=8)	Many trials incorporated EHR systems in the intervention packages, including three scenarios: (1) electronic reminders or alerts to health providers based on EHR data, (2) clinical decision support systems built in the EHR system, and (3) monitoring of EHR data by health providers to make clinical decisions.	Strength: EHR could integrate different intervention components of complex interventions. Strength: the EHR-based data review by health providers allowed real-time monitoring of patients’ health. Limitation: lack of complete and high-quality data in EHR systems would prohibit relevant, timely, and accurate clinical decision support. Limitation: technical glitches such as server breakdown compromised the continuity of EHR-based interventions.	[16,19,21,23,38,40,44]
Fidelity (n=2)	Some trials used EHR systems to assess the fidelity of trial conduct according to predefined protocols, such as providers’ adherence to study protocols and patients’ adherence to lifestyle changes.	Not mentioned.	[19,40]
Primary outcome assessment (n=24)	Most included trials used EHR systems to assess studies’ primary outcomes, including clinical data (eg, blood pressure), behavioral data (eg, medication use), and service use data (frequency of hospital visits).	Strength: using EHR for data extraction is time saving compared with conventional individual chart review. Limitation: EHR’s data quality could be questionable, subject to inaccuracy, misclassification, and incompleteness. Limitation: some relevant information was not available in the EHR systems. Limitation: lack of integration across or linkage with different EHR systems might cause missing information or underdetection of events. Limitation: EHR was not able to provide information for individuals who moved out of the region in the middle of the trial. Limitation: some data might be missing or omitted in the transformation from handwritten medical records to EHR.	[17,21-25,27-38,40-43,45,46]
Nonprimary outcome assessment (n=13)	Many trials used EHR systems to assess studies’ nonprimary outcomes, including clinical, behavioral, and service use data. In addition, 1 study used EHR to assess safety outcomes (eg, the occurrence of adverse events).	Strength: using EHR for data extraction is time saving compared with conventional individual chart review. Limitation: some relevant information was not available in the EHR systems, which might omit potential confounders.	[17,21,22,25,33,35-42]
Extended follow-up (n=2)	A few trials used EHR systems to follow-up with participants beyond the study time frame to determine the sustainability of effects.	Not mentioned.	[21,35]

^a^EHR: electronic health record.

We found 8 studies that incorporated EHR into their intervention packages. There were 3 types of interventions that used EHR. First, 3 studies used electronic reminders or alerts to health care providers based on EHR data [19,40,44]. For example, Puttkammer et al [40] used the EHR systems to alert physicians of patients at elevated risk of treatment failure through automated calculations of patients’ risk score based on their past EHR data. Second, 4 studies incorporated clinical decision support systems into the EHR systems [16,21,25,38]. Third, 2 studies involved manual monitoring and review of EHR data by health care providers [23,38]. For example, Ali et al [38] included both an EHR-based clinical decision support system and manual monitoring of EHR data. Their EHR-based clinical decision support system integrated patient characteristics, depressive symptom scores, and laboratory data to provide evidence-based guidelines to physicians based on treatment guidelines, and the study team also manually monitored and reviewed the EHR data and developed consensus recommendations for patients with severe symptoms.

A total of 6 studies used EHR to collect baseline information, which mainly included individual demographic, socioeconomic, and clinical information to determine the basic characteristics of the participants. For example, 1 study collected baseline information through EHRs, including age, sex, pregnancy status, HIV clinic enrollment date, and antiretroviral treatment initiation date among people with HIV or AIDS in Zambia [43].

Only 2 studies used EHR for fidelity assessment of the trial. Puttkammer et al [40] used EHR to determine health workers’ “engagement” with the EHR-based computerized alert, which was defined as the frequency of the health workers’ clicking on the alert to bring up the “pop up” window. Figar et al [19] used EHR to determine adherence to lifestyle changes in older patients with hypertension [19]. Both studies were feasibility studies and both lacked randomization.

Two studies used EHR to follow-up with participants beyond the study timeframe to determine the sustainability of the effects [21,35]. Phillips et al [35] used EHR to follow-up with female participants who attended a past trial on HIV or AIDS to determine the continued effects of the interventions on female participants’ retention in HIV care and viral suppression. Were et al [21] queried the EHR data 3 months after the study closure to capture the study’s sustained effects on the quality of pediatric HIV care in a resource-limited setting in Kenya.

Some studies discussed the strengths and limitations of using EHR in conducting clinical trials. For participant identification and recruitment, Semeere et al [18] reported that EHR enabled researchers to access participants who were otherwise unfeasible to access, and Bachmann et al [30] mentioned that EHR enabled researchers to identify and include all eligible patients whose interactions with health systems were electronically recorded, which increased generalizability and sample size at minimal cost. However, a few studies shared concerns about the inclusion of ineligible participants or exclusion of eligible participants owing to artifacts, errors, and misclassifications in EHR [20,30,33].

For studies that incorporated EHR in their interventions, Ali et al [38] mentioned the advantage of EHR to “integrate different intervention components of complex interventions,” and Zhang et al [23] mentioned that EHR systems enabled real-time monitoring and reviewing of patients’ health data by health providers. For limitations, by contrast, Were et al [21] argued that the lack of complete and high-quality data in EHR systems prohibits relevant, timely, and accurate clinical decision support. The server breakdown in the study by Puttkammer et al [40] represented a general concern about potential technical glitches of EHR, which could lead to risks of discontinuity of interventions.

For studies that used EHR for outcome assessment, an important strength was that it was more time saving than conventional outcome research approaches, for which databases and patient registries are often fragmented and limited in the number of patients [24]. However, numerous limitations were documented, particularly concerning data quality. These limitations included inaccuracies, misclassifications, and incompleteness, which were evident in various aspects, including the use of EHR for participant identification, recruitment, baseline information collection, and interventions [33,35,43]. Data quality issues were emphasized in the study by Oluoch [24], which observed a loss of information in the transformation process from handwritten records to EHR. However, 1 study argued that these intrinsic flaws in EHR data represented the situation of real-world care delivery and was thus valuable in its own way [43]. Four studies mentioned that not all relevant information was available in EHR systems, which could prohibit suboptimal data analysis such as underadjustment for study confounders [34,36,40,43]. Finally, some studies mentioned that the lack of integrated EHR systems across different health facilities might cause missing information or underdetected events [35,42,44].

### Roles of EHRs in Trial Designs

**Figure 3:**
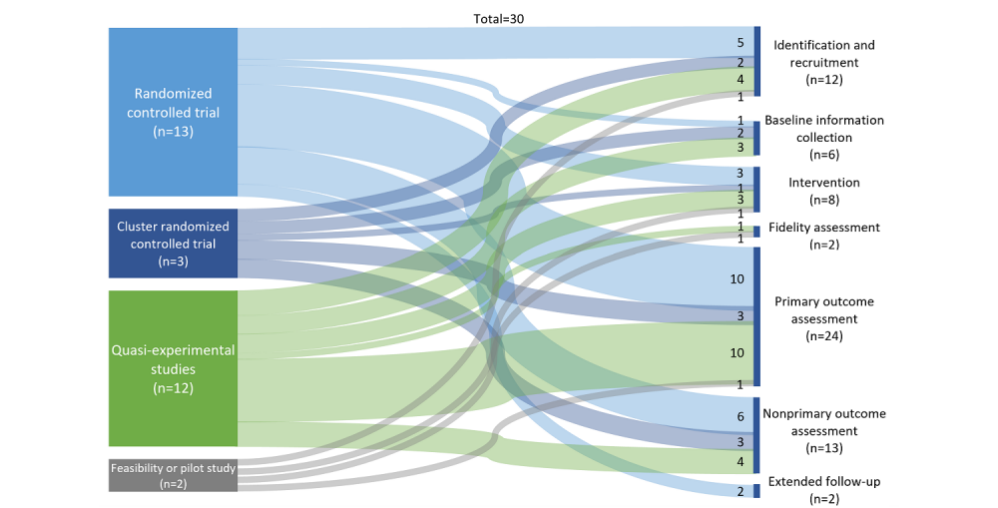
The roles of electronic health records in conducting clinical trials by different trial designs.

We further found that the roles of EHR in conducting clinical trials differed by different trial designs (Figure 3). Of the 4 types of trials, RCT covered the most steps of conducting trials that used EHR and was the only type that used EHR for “extended follow-up” [21,35]. Other than that, RCTs, cluster RCTs, and quasi-experimental studies covered the same 5 steps of conducting trials using EHR: participant identification and recruitment, baseline information collection, intervention, primary and nonprimary outcome assessment. Notably, a controlled trial without randomization [19] and a feasibility study [40] were the only 2 studies that used EHR to assess the fidelity of conducting their interventions.

### Quality Assessment

The quality assessment results are presented in Multimedia Appendices 2 and 3. Less than half of the included studies were found to be of good quality (14/30, 47%), 40% (12/30) of fair quality, and 13% (4/30) of poor quality. For the 20 studies that had control groups (ie, all the RCTs, cluster RCTs, non-RCTs, some quasi-experimental studies, and 1 of the feasibility pilot studies), the most common factors that compromised the quality of the studies was the lack of prespecification of outcomes and subgroups (9/30, 30%), followed by the lack of similar baseline characteristics between groups (8/30, 27%), lack of blinding in treatment assignment (7/30, 23%) and outcome assessment (7/30, 23%), and nonreport of calculations for sufficient sample size (7/30, 23%). Of the 10 studies that did not have control groups, 7 (70%) did not have interrupted time series design, 6 (60%) did not report sufficient sample size, and 5 (50%) had suboptimal representativeness of the participants to the target population.

## Discussion

### Summary of Principal Findings

We synthesized our findings on the use of EHR for conducting clinical trials in LMICs into a framework that depicts the typical life cycle of a clinical trial. The EHRs were primarily used for eligible participant identification or recruitment, trial outcome assessment, and intervention implementation in LMICs. The limited use of EHR was documented for participant consent, randomization, and fidelity assessment. An encouraging observation was the diversity of disease types covered in the selected studies, highlighting that EHRs have a wide appeal across various medical domains. Notably, a wide range of outcomes were assessed using EHRs in these trials, including clinical outcomes, behavioral outcomes, and health service outcomes.

### Characteristics of EHR-Supported Trials in LMICs

The identified EHR-supported trials in LMICs were heterogeneous in terms of their targeted populations and outcomes. LMICs include, by nature, a diverse group of regions with varying population characteristics including health conditions and socioeconomic status. In general, many LMICs reportedly face challenges in terms of technological infrastructure [47], data quality, and interoperability of EHR systems, which can impact the feasibility and reliability of EHR-based trials [48]. Moreover, regulatory frameworks and guidelines for EHR-supported clinical trials have been poorly defined in some LMICs, especially for data use and security in these settings [49].

In our review, although there was a clear use of EHR in conducting clinical trials in LMICs, few of them focused on medication. One possible explanation is that the focus of the medication-related trials is more explanatory (ie, understanding whether an intervention or medication is efficacious in an ideal setting) rather than pragmatic (ie, determining the effectiveness of interventions in real-world settings). As EHRs are usually routine health information systems rather than tools specialized for clinical trials, they are naturally more suitable for studies focusing on real-world effectiveness and implementation of an intervention but not necessarily for those focusing on intervention efficacy under strictly controlled conditions. In addition, the reported data quality issues and lack of population coverage in EHRs may also have limited their usability in efficacious studies. However, EHR can still be used to inform planning, participant recruitment, baseline statistics, and outcome extraction in medication-related trials [41]. Given that current medication development studies are primarily conducted in HICs, future uses of EHR may have the potential to enable more medication-related trials to be conducted in LMICs, thus increasing the representation of LMICs in study populations and geographic settings at a relatively low cost [50,51].

### Challenges of Using EHRs in Clinical Trials in LMICs

There are 2 potential challenges to using EHRs in clinical trials. The first relates to possible barriers, including difficulties in accessing relevant data, linking different data sources, high financial costs, and limited familiarity with such systems. The second pertains to the underreporting or exclusion of EHR information. For example, researchers may neglect the inclusion of EHR data owing to time constraints or competing priorities [52] or selectively report the EHR data that aligns with their hypotheses or desired outcomes, potentially introducing bias into the results [53]. Integrating EHR data into a clinical trial can be technically complex and time consuming [54]. Researchers may lack the resources or technical expertise to effectively integrate EHR data with the clinical trials’ data set, leading to the decision to exclude or underreport it [12,55].

Another prominent observation was that almost all the included articles expressed challenges with using EHR, with some being explicit experiences and lessons. Common challenges were typically related to data availability, data quality, data interoperability, and missing data. For data availability, it meant that some relevant components to define a clinical entity were absent [35]. For data quality, the most common concern was data missingness, such as missing laboratory values in an EHR system; data artifacts were also a main concern, especially when the data were manually entered into the EHR [22]. Ultimately, the specifics of these challenges can be potentially beneficial for developing guidance on optimal EHR uses [56]. Specific to the LMICs, a tailed framework for using EHRs in clinical trials may be useful to assess the fitness of EHR for the trials [57]; using the insights from these identified challenges may be useful in ensuring the EHR selected best fits the desired need [58,59].

None of the selected studies used EHRs to collect participants’ consent information and conduct randomization, which has been successfully performed in HICs. For example, the *Join Us* initiative in Australia uses the linkage of the routinely collected data including EHRs to recruit residents and collect their consent to enroll in potential clinical trials [60]. To do this in LMICs, it may be necessary to establish an updated regulatory framework for research ethics, such as the consent process for using and sharing routinely collected data and for intervention implementation. Nevertheless, obtaining informed consent for automated trials conducted using EHRs may be difficult [5]. For example, there is still debate on whether informed consent needs to be acquired when only variations of usual care are explored [61]. There are also disconnections between clinical trials that use EHRs and regulation guidelines created for traditional RCTs without the involvement of EHRs (eg, the lack of standardized requirements of institutional review boards for the use of EHRs in trials). With more examples of EHR-supported trials emerging, further research and constructive dialogues among all stakeholders are needed to alter and align the ethical norms and regulatory processes to enable more successful and accountable uses of EHRs in clinical trials in LMICs.

Notably, the quality assessment of the included trials indicated that a substantial portion of the studies did not meet the criteria for good quality. The lack of prespecification of outcomes and subgroups, for example, emerged as a notable issue, which may lead to outcome reporting bias and ambiguity in result interpretation. Other major issues included the absence of comparable baseline characteristics between the treatment and control groups, inadequate blinding, lack of considerations for sample size sufficiency, and lack of control groups. On the one hand, these commonly identified shortcomings revealed the exploratory nature of many of the included EHR-supported trials in LMICs, which needs to be addressed in future efforts to enhance rigor and credibility. On the other hand, they also implied that quasi-experimental designs, such as interrupted time series and self-controlled studies, might be the “comfort zone” for using EHRs to support future clinical trials.

### Limitations

This scoping review has some limitations. First, we only focused on EHRs in clinical trials in the context of LMICs, rather than comparing the results between HICs and LMICs. Different LMICs follow different data schemas and regulatory structures, which may lead to challenges when considering generalizability. Second, we required the mention or self-tagging of EHR within the articles. This requirement likely led to a swath of missed potential articles. However, we included a wide range of synonyms of EHR in the search syntax, which should have helped address this limitation. Third, we did not test any hypothesis regarding the effect of using EHR in clinical trials, and we did not we assess the impact of using EHRs on health outcomes. Although we extracted a few characteristics that could point to the methodological quality of the studies, including the evaluation of risk of bias, we did not evaluate the intervention effects reported in the trials but merely offered a description of EHRs’ roles in the trial conduct.

### Conclusions

We mapped the roles of EHRs in clinical trials from the selected studies to the life cycle of clinical trials and identified opportunities to enhance the use of EHRs for clinical trials in LMICs. Specifically, the most commonly documented use was the incorporation of EHRs into clinical trials for outcome assessment, whereas the use of EHR in collecting participant consent and conducting randomization was scarce. Efforts should be made to improve the curation of EHR data to improve data quality, explore the integration of automated processes in EHR to obtain people’s consent for data use in research, and standardize regulatory frameworks for using EHR for research. Future research and practices are recommended to navigate the strengths of EHRs, such as time sensitivity and low costs, and mitigate the current challenges, such as suboptimal data quality and limited population coverage, to ensure better use of EHR in future clinical trials in LMICs. With the ongoing digitization of health information systems globally, researchers, practitioners, and policy makers are recommended to maintain continued evaluations of the availability and quality of EHRs to better understand their optimal use and unlock the full potential of EHRs for health care services and research purposes.

## Appendix

Multimedia Appendix 1 Search syntax by each database.

Multimedia Appendix 2 Quality assessment of included trials with independent control groups (part A).

Multimedia Appendix 3 Quality assessment of included trials without independent control groups (part A).

Multimedia Appendix 4 PRISMA-ScR-Checklist.

## Abbreviations

EHR: electronic health record
HIC: high-income country
LMIC: low- and middle-income countries
PICOS: patients, problem, or population; issue of interest or intervention; comparison, control, or comparator; outcome; and study type
PRISMA: Preferred Reporting Items for Systematic Reviews and Meta-Analyses
PRISMA-ScR: Preferred Reporting Items for Systematic Reviews and Meta-Analyses extension for Scoping Reviews
RCT: randomized controlled trial

## Data Availability

The authors declare that all the data included in this study are available in the paper and the multimedia appendices.
