# Correction: The Roles of Electronic Health Records for Clinical Trials in Low- and Middle-Income Countries: Scoping Review

**DOI:** 10.2196/84394

**Published:** 2025-10-08

Following the publication of “The Roles of Electronic Health Records for Clinical Trials in Low- and Middle-Income Countries: Scoping Review” [1], concerns were raised regarding the author Jiancheng Ye’s rate of self-citation within the article’s reference list. Further investigation into this matter identified that numerous self-citations were added to the manuscript following acceptance of the article without adequate disclosure.

All authors were contacted for a response regarding this matter. Authors Shangzhi Xiong, Tengyi Wang, Jingyi Li, Nan Cheng, Maoyi Tian, and Yang Yang indicated that they were unaware of the addition of references post acceptance. They did not consent to such changes and believed a number of the additions to be irrelevant to the content of the article and requested their removal. Author Jiancheng Ye responded with a scientific justification for each added self-citation within the published article.

Following review of these responses, the JMIR Publications Editorial Office determined that several of the added references were not relevant to the article or were used to support a generic statement where another reference could have been used instead of self-citation. Therefore, the JMIR Publications Editorial Office is issuing this corrigendum to remove the following references:

- Reference 13: Ye J, Wang Z, Hai J. Social networking service, patient-generated health data, and population health informatics: national cross-sectional study of patterns and implications of leveraging digital technologies to support mental health and well-being. J Med Internet Res 2022 Apr 29;24(4):e30898 [doi: 10.2196/30898] [Medline: 35486428]
- Reference 14: Ye J. The role of health technology and informatics in a global public health emergency: practices and implications from the COVID-19 pandemic. JMIR Med Inform 2020 Jul 14;8(7):e19866 [doi: 10.2196/19866] [Medline: 32568725]
- Reference 48: Ye J, Orji IA, Baldridge AS, Ojo TM, Shedul G, Ugwuneji EN, et al. Characteristics and patterns of retention in hypertension care in primary care settings from the hypertension treatment in Nigeria program. JAMA Netw Open 2022 Sep 01;5(9):e2230025 [doi: 10.1001/jamanetworkopen.2022.30025] [Medline: 36066896]
- Reference 49: Ye J, Li N, Lu Y, Cheng J, Xu Y. A portable urine analyzer based on colorimetric detection. Anal Methods 2017;9(16):2464-2471 [doi: 10.1039/C7AY00780A]
- Reference 51: Ye J, Ren Z. Examining the impact of sex differences and the COVID-19 pandemic on health and health care: findings from a national cross-sectional study. JAMIA Open 2022 Sep 28;5(3):ooac076 [doi: 10.1093/jamiaopen/ooac076] [Medline: 36177395]
- Reference 52: Ye J, Yao L, Shen J, Janarthanam R, Luo Y. Predicting mortality in critically ill patients with diabetes using machine learning and clinical notes. BMC Med Inform Decis Mak 2020 Dec 30;20(Suppl 11):295 [doi: 10.1186/s12911-020-01318-4] [Medline: 33380338]
- Reference 53: Ye J. Pediatric mental and behavioral health in the period of quarantine and social distancing with COVID-19. JMIR Pediatr Parent 2020 Jul 28;3(2):e19867 [doi: 10.2196/19867] [Medline: 32634105]
- Reference 54: Ye J. Advancing mental health and psychological support for health care workers using digital technologies and platforms. JMIR Form Res 2021 Jun 30;5(6):e22075 [doi: 10.2196/22075] [Medline: 34106874]
- Reference 55: Ye J, Hai J, Wang Z, Wei C, Song J. Leveraging natural language processing and geospatial time series model to analyze COVID-19 vaccination sentiment dynamics on Tweets. JAMIA Open 2023 Apr 12;6(2):ooad023 [doi: 10.1093/jamiaopen/ooad023] [Medline: 37063408]
- Reference 59: Ye J. Patient safety of perioperative medication through the lens of digital health and artificial intelligence. JMIR Perioper Med 2023 May 31;6:e34453 [doi: 10.2196/34453] [Medline: 37256663]
- Reference 62: Ye J, Woods D, Bannon J, Bilaver L, Kricke G, McHugh M, et al. Identifying contextual factors and strategies for practice facilitation in primary care quality improvement using an informatics-driven model: framework development and mixed methods case study. JMIR Hum Factors 2022 Jun 24;9(2):e32174 [doi: 10.2196/32174] [Medline: 35749211]

The JMIR Publications Editorial Office regrets that these issues were not identified prior to publication.

The correction will appear in the online version of the paper on the JMIR Publications website together with the publication of this correction notice. Because this was made after submission to PubMed, PubMed Central, and other full-text repositories, the corrected article has also been resubmitted to those repositories.
