# Supplementary material for: The Roles of Electronic Health Records for Clinical Trials in Low- and Middle-Income Countries: Scoping Review
Source: JMIR Med Inform. 2023 Nov 22;11:e47052. doi: 10.2196/47052 (PMC10701650; doi:10.2196/47052)
Supplement: Multimedia Appendix 1 [file medinform_v11i1e47052_app1.pdf]

Appendix 3: Search syntax by each database

(The LMIC filters came from: https://epoc.cochrane.org/lmic-filters)

1. **Embase**

health data/ or administrative health data/ or exp medical record/ or routinely collected health data/ or (routinely collected data or electronic medical record* or electronic health record* or computerized health record* or computerized health data or electronic health data or electronic healthcare data or electronic healthcare record* or electronic health care record* or electronic health care data or e-health record* or personal health record* or real-world data or real-world evidence or electronic patient reported outcome*).ti,ab,kw.
AND exp human experiment/ or exp clinical trial/ or exp "clinical trial (topic)"/ or exp intervention study/ or exp pilot study/ or exp feasibility study/ or (clinical trial* or controlled trial* or pragmatic trial* or intervention* stud* or intervention* trial* or experiment* stud* or experiment* trial* or pilot stud* or feasibility stud* or multi-arm multi-stage trial*).ti,ab,kw.
AND LMIC filter

2. **Pubmed**

"Electronic Health Records"[MeSH Terms] OR "health records, personal"[MeSH Terms] OR "medical records systems, computerized"[MeSH Terms] OR "Routinely Collected Health Data"[MeSH Terms] OR "Health Information Exchange"[MeSH Terms] OR "Health Information Systems"[MeSH Terms] OR routinely collected data[Text Word] OR electronic medical record*[Text Word] OR electronic health record*[Text Word] OR computerized health record*[Text Word] OR computerized health data[Text Word] OR electronic health data[Text Word] OR electronic healthcare data[Text Word] OR electronic healthcare record*[Text Word] OR electronic health care record*[Text Word] OR electronic health care data[Text Word] OR e-health record*[Text Word] OR personal health record*[Text Word] OR real-world data[Text Word] OR real-world evidence[Text Word] OR electronic patient reported outcome*[Text Word]
AND "Clinical Trials as Topic"[MeSH Terms] OR "Clinical Trial"[Publication Type] OR "Pilot Projects"[MeSH Terms] OR "Feasibility Studies"[MeSH Terms] OR "clinical trial*"[Text Word] OR "controlled trial*"[Text Word] OR "pragmatic trial*"[Text Word] OR "intervention stud*"[Text Word] OR "intervention trial*"[Text Word] OR "experiment stud*"[Text Word] OR "experiment trial*"[Text Word] OR "pilot stud*"[Text Word] OR "feasibility stud*"[Text Word] OR "multi arm multi stage trial*"[Text Word]
AND LMIC filter

3. **CINAL**

(MH "Patient Record Systems+") OR (MH "Electronic Health Records+") OR (MH "Medical Records, Personal") OR (MH "Medical Records+") OR (MH "Routinely Collected Health Data") OR TX routinely collected data or electronic medical record* or electronic health record* or computerized health record* or computerized health data or electronic health data or electronic healthcare data or electronic healthcare record* or electronic

health care record* or electronic health care data or e-health record* or personal health record* or real-world data or real-world evidence or electronic patient reported outcome*
AND (MH "Experimental Studies+") OR (MH "Clinical Trials+") OR (MH "Randomized Controlled Trials+") OR (MH "Pretest-Posttest Design+") OR (MH "Pilot Studies") OR TX clinical trial* or controlled trial* or pragmatic trial* or intervention* stud* or intervention* trial* or experiment* stud* or experiment* trial* or pilot stud* or feasibility stud* or multi-arm multi-stage trial*
AND LMIC filter

4. SCOPUS

TITLE-ABS-KEY ("routinely collected data" OR "electronic medical record*" OR "electronic health record*" OR "computerized health record*" OR "computerized health data" OR "electronic health data" OR "electronic healthcare data" OR "electronic healthcare record*" OR "electronic health care record*" OR "electronic health care data" OR "e-health record*" OR "personal health record*" OR "real-world data" OR "real-world evidence" OR "electronic patient reported outcome*")
AND TITLE-ABS-KEY ("clinical trial*" or "controlled trial*" or "pragmatic trial*" or "intervention* stud*" or "intervention* trial*" or "experiment* stud*" or "experiment* trial*" or "pilot stud*" or "feasibility stud*" or "multi-arm multi-stage trial*")
AND LMIC filter

5. Cochrane

(routinely collected data or electronic medical record* or electronic health record* or computerized health record* or computerized health data or electronic health data or electronic healthcare data or electronic healthcare record* or electronic health care record* or electronic health care data or e-health record* or personal health record* or real-world data or real-world evidence or electronic patient reported outcome*):ti,ab,kw
AND (clinical trial* or controlled trial* or pragmatic trial* or intervention* stud* or intervention* trial* or experiment* stud* or experiment* trial* or pilot stud* or feasibility stud* or multi-arm multi-stage trial*):ti,ab,kw
AND LMIC filter
