# Supplementary material for: The Roles of Electronic Health Records for Clinical Trials in Low- and Middle-Income Countries: Scoping Review
Source: JMIR Med Inform. 2023 Nov 22;11:e47052. doi: 10.2196/47052 (PMC10701650; doi:10.2196/47052)
Supplement: Multimedia Appendix 2 [file medinform_v11i1e47052_app2.pdf]

Appendix 2. Quality assessment of included trials with independent control groups (Part A)

Study	Randomized controlled trial?	Adequate randomization?	Concealed treatment allocation?	Blinded treatment assignment?	Blinded outcome assessment?	Similar baseline characteristics between groups?	Low drop-out rates?
Silvana Figar, 2004	0	0	0	0	0	0	1
Najla A. Lakkis, 2011	1	1	1	1	na	na	1
Martin C. Were, 2013	1	1	1	1	na	1	1
Yuan Li, 2014	0	0	na	na	na	na	na
Alexandra S. Ghadieh, 2015	1	1	1	1	na	na	1
Mohammed K. Ali, 2016	1	1	1	0	0	0	1
Tom Oluoch, 2015	1	1	0	1	0	0	1
Zengwu Wang, 2016	0	0	na	na	na	0	0
Amna Al-Hashar, 2018	1	1	1	0	1	1	1
Gavin George, 2018	1	na	na	1	na	na	1
Max Oscar Bachmann, 2019	0	1	0	0	0	0	1
Elizabeth A. Kelvin, 2018	1	na	na	1	0	1	1
Tamsin K Phillips, 2020	1	na	na	0	1	1	1
Mohammed K. Ali, 2020	1	1	1	0	1	1	1
Guoyong Yang, 2020	1	1	1	1	1	1	1
Nancy Puttkammer, 2020	0	0	0	na	na	0	1
Monika Roy, 2020	1	1	0	0	0	1	1
Seth Kalichman, 2020	1	1	1	na	1	1	1
Abda Mahmood, 2020	1	0	na	1	1	0	1
Surajudeen Abiola Abdulrahman , 2017	1	1	1	1	0	0	1

Appendix 2. Quality assessment of included trials with independent control groups (Part B)

Study	Similar drop-out rates between groups?	Adherence to intervention protocols?	Similar background treatments?	Valid and reliable measures?	Sufficient sample size?	Pre-specified outcomes and subgroups?	Intention-to-treat analysis?
Silvana Figar, 2004	1	1	1	1	1	0	1
Najla A. Lakkis, 2011	1	1	1	1	0	0	1
Martin C. Were, 2013	1	1	1	1	0	1	1
Yuan Li, 2014	na	1	1	1	0	0	1
Alexandra S. Ghadieh, 2015	1	1	1	1	1	0	1
Mohammed K. Ali, 2016	1	1	1	1	1	1	1
Tom Oluoch, 2015	1	1	1	1	1	1	1
Zengwu Wang, 2016	na	1	1	1	0	0	1
Amna Al-Hashar, 2018	1	1	1	1	1	1	1
Gavin George, 2018	1	1	1	1	0	0	1
Max Oscar Bachmann, 2019	1	1	1	1	1	1	1
Elizabeth A. Kelvin, 2018	1	1	1	1	1	1	1
Tamsin K Phillips, 2020	1	1	1	1	0	0	1
Mohammed K. Ali, 2020	1	1	1	1	1	1	1
Guoyong Yang, 2020	1	1	1	1	1	1	1
Nancy Puttkammer, 2020	1	1	1	1	0	0	1
Monika Roy, 2020	1	1	1	1	1	1	1
Seth Kalichman, 2020	1	1	1	1	1	0	1
Abda Mahmood, 2020	1	1	1	1	1	1	1
Surajudeen Abiola Abdulrahman , 2017	1	1	1	1	1	1	1
