# Supplementary material for: The Roles of Electronic Health Records for Clinical Trials in Low- and Middle-Income Countries: Scoping Review
Source: JMIR Med Inform. 2023 Nov 22;11:e47052. doi: 10.2196/47052 (PMC10701650; doi:10.2196/47052)
Supplement: Multimedia Appendix 3 [file medinform_v11i1e47052_app3.pdf]

Appendix 3. Quality assessment of included trials without independent control groups (Part A)

Study	Clearly stated objectives?	Clearly described eligibility criteria of study population?	Representativeness of participants?	Inclusiveness of all eligible participants?	Sufficient sample size?	Intervention clearly described and consistently delivered?
Hai-xia Zhang, 2014	1	1	0	1	0	1
Aaloke Mody, 2018	1	1	0	1	0	1
Riekie Engelbrecht, 2018	1	1	0	1	0	1
Sherine Ismail, 2019	1	1	0	1	1	1
Mariana F. Lima, 2018	1	1	1	na	0	1
Kim Heng Tay, 2019	1	1	0	1	1	1
Xinbao Wu, 2019	1	1	1	1	0	1
Aaloke Mody, 2021	1	1	1	1	1	1
Aggrey Semeere, 2021	1	1	1	1	1	1
Shanshan Xu, 2021	1	1	1	1	0	1

Appendix 3. Quality assessment of included trials without independent control groups (Part B)

Study	Pre-specified, valid and reliable measures?	Blinded outcome assessment?	Low loss-to follow-ups?	P values provided for before-after comparisons?	Interrupted time-series design?	Individual-level data accounted for in collective analyses?
Hai-xia Zhang, 2014	1	1	1	1	0	na
Aaloke Mody, 2018	1	na	0	1	1	na
Riekie Engelbrecht, 2018	1	na	1	1	0	na
Sherine Ismail, 2019	1	0	1	1	1	na
Mariana F. Lima, 2018	1	na	na	1	0	na
Kim Heng Tay, 2019	1	na	1	1	0	na
Xinbao Wu, 2019	1	na	1	1	0	na
Aaloke Mody, 2021	1	na	na	1	1	1
Aggrey Semeere, 2021	1	0	1	0	0	na
Shanshan Xu, 2021	1	0	1	1	0	na
